# Protective Role of UCP2 in Oxidative Stress and Apoptosis during the Silent Phase of an Experimental Model of Epilepsy Induced by Pilocarpine

**DOI:** 10.1155/2018/6736721

**Published:** 2018-08-06

**Authors:** Marina Rascio Henriques Dutra, Regiane dos Santos Feliciano, Kalil Ribeiro Jacinto, Telma Luciana Furtado Gouveia, Eduardo Brigidio, Andrey Jorge Serra, Mariana Morris, Maria da Graça Naffah-Mazzacoratti, José Antônio Silva

**Affiliations:** ^1^Universidade Nove de Julho, Rua Vergueiro 249, 01504-001 São Paulo, SP, Brazil; ^2^Universidade Federal de São Paulo, Rua Pedro de Toledo 697, 04039-001 São Paulo, SP, Brazil; ^3^Nova Southeastern University, 3301 College Ave, Fort Lauderdale, FL 3314, USA

## Abstract

Neuroprotection is a desirable process in many neurological disorders, yet complex mechanisms involved in this field are not completely understood. The pilocarpine epilepsy model causes potent, seizure-induced excitotoxicity cell death and mitochondria impairment. The present study is aimed at investigating the role of UCP2, a ROS negative regulator, in the neuroprotection after cholinergic insult. Our data demonstrated that UCP2 expression was augmented in the rat hippocampus 3 days after *status epilepticus* (SE), reaching a peak on the fifth day, then returning to basal levels. Concomitantly, phospho-AKT expression levels were higher in the hippocampus during the early silent phase (5 days after SE). Additionally, it was demonstrated that the blockade of UCP2 by antisense oligonucleotides (ASO) in SE rats successfully diminished both UCP2 mRNA and protein contents. SE ASO rats presented increased mitochondrial proapoptotic factor expression, caspase-3 activity, inflammatory cytokine expression, and ROS formation. Moreover, ASO treatment diminished p-AKT expression and antioxidant enzyme activities after pilocarpine insult. In conclusion, the present results highlight the neuroprotective actions of UCP2, acting in the inhibition of apoptotic factors and oxidative stress, to increase neuron survival after SE onset.

## 1. Introduction

Mitochondria are energy-producing organelles widely involved in cell homeostasis maintenance and have been described as a potential site for the intricate events that result in pathological disorders and cell death. The uncoupling proteins (UCPs) are anion-carrier proteins found in the inner membrane of the mitochondria and are involved in diminishing the transmembrane proton gradient [1]. This activity reduces the drive for ROS production and consequently decreases cell death [2, 3]. Thus far, five UCP isoforms have been described based on their sequence homology with UCP1 and their distinct functions [4].

UCP2 is one member of this family and is widely expressed in neurons and immune cells [5–7]. Increased UCP2 expression was observed in immune and nonimmune cells during pathological states such as atherosclerosis [8], type I diabetes [9], infections [10], cerebral ischemia [11], and experimental autoimmune encephalomyelitis (EAE) [12]. Several pathophysiological conditions might generate stimuli that can lead to increased UCP2 expression, resulting in a neuroprotection process. UCP2 mitigates reactive oxygen species (ROS) production, therefore protecting these cells from the damage of oxidative stress [2, 5, 13]. UCP2 has been implicated in intracellular calcium regulation, ATP production, synaptic transmission, neuronal plasticity, and apoptosis [5, 6, 14, 15].

Inflammatory signaling activation is an important mechanism leading to augmented ROS production and, consequently, to incremental cell death in several cell types, including neurons [16–18]. In experimental epilepsy, after the insult that generates seizures, a potent inflammatory state and significant neuronal death can be observed [19–21]. UCP2 is considered as an important neuroprotective element in many inflammatory and degenerative states of the central nervous system [22], especially for its ability to decrease reactive oxygen species [12].

For more than a decade, our group has been dedicated in studying the role of inflammation in the pathophysiology of epilepsy, which is supported by the findings that interleukins and vasoactive peptide system (kallikrein-kinin and renin-angiotensin) components have distinct actions protecting or worsening seizure activity in both mesial temporal lobe epilepsy (MTLE) patients and experimental animal models [23–26]. The acute administration of high-dose of pilocarpine in rats is an experimental model that has revealed alterations that are comparable to those in human TLE (for review, see [27]). Besides that the mechanism of pilocarpine-induced neurotoxic seizures is well established, it is presently hypothesized that excitotoxicity inflicted by *status epilepticus* (SE) induced by pilocarpine results in pathological increases in neuronal lesions in response to excessive ROS production [23, 24, 26].

To our knowledge, few studies show a relation between seizure activity and UCP2 expression in epilepsy or its implication in apoptosis activation [28–31]. In the present study, we tested the hypothesis that UCP2 can act as an endogenous protective factor against epilepsy-induced damage, using antisense oligonucleotide (ASO) administration, in an experimental pilocarpine model.

## 2. Methods

### 2.1. Experimental Groups

We used thirty male Wistar rats (200–250 g) that were randomized into 3 experimental groups. The experimental protocol was approved by the Committee on the Ethics of Animal Experiments of Universidade Nove de Julho (0034/2012). Rats were anesthetized with intraperitoneal (ip) sodium pentobarbital (50 mg/kg), and then the animals received a single dose of pilocarpine (350 mg/kg, ip). To prevent peripheral cholinergic effects, scopolamine methyl nitrate was injected subcutaneously at a dose of 1 mg/kg, 30 min before pilocarpine administration. A group of animals (*n* = 5) was killed 5 h after *status epilepticus* onset (5 h SE, the acute group). Another group (*n* = 5) was killed during the seizure-free period (5 days after SE onset, the silent group), and the last set (*n* = 5) was killed 60 days after SE induction (period of spontaneous recurrent seizures, the chronic group). Saline-treated animals (*n* = 15) were killed 5 h, 5 days, or 60 days after saline and scopolamine methyl nitrate injections and were used as control (control groups). Seizures were observed and scored using the Racine scale [32] for 4 h and then rats received a 4 mg/kg dose of diazepam to terminate SE. To verify UCP2 expression in a time course protocol through the three phases of the pilocarpine model, we used 25 Wistar male rats that were anesthetized and randomly killed at 1, 5, and 24 hours and 3, 5, 7, 45, and 90 days after pilocarpine administration.

All investigation followed the university guidelines for the use of animals in experimental studies, and all efforts were made to minimize suffering, conformed to the *Guide for the Care and Use of Laboratory Animals* published by the US National Institutes of Health (NIH publication number 85–23, revised 1996). The animals were kept on a 12 : 12 h artificial light : dark cycle with rodent chow and water provided ad libitum. All samples were used to perform mRNA-, protein-, or ROS-related compounds quantification.

### 2.2. Immunohistochemistry

SE rats (5 hours (5H), 5 days (5D), sixty days (60D), and their “control”) were anesthetized with a lethal sodium dose of pentobarbital and subjected to transcardiac perfusion with a solution of paraformaldehyde 1% (pH 7.4, 15 mL/rat, infusion rate 15 mL/minute) followed by a solution of paraformaldehyde 4% (pH 7.4, 150 mL/rat, infusion rate 15 mL/minute). After perfusion, the brain was carefully detached from the skull, fixed in paraformaldehyde 4% for 48 hours, and immersed in a solution of sucrose 30% for cryoprotection for 48 hours. Forty-micrometer-thick coronal slices were obtained using a cryostat (HM 505E Micromeria, Zeiss) and stored in 0.1 M phosphate buffer (pH 7.4). The slices were collected throughout the hippocampus and stored in 0.1 M phosphate buffer. The slices were mounted on gelatin-coated slides for immunohistochemistry with p-AKT antibody (Santa Cruz, 1 : 200). Briefly, free-floating slices were treated with hydrogen peroxide 1% for 10 minutes, washed with phosphate-buffered saline (PBS) (pH 7.4), and then treated with Triton X-100 0.4% for 30 minutes. Slices were washed with PBS, preincubated with albumin 10% for 2 hours, and incubated with primary antibody overnight at 4°C. The slices were washed and then incubated at room temperature with appropriate secondary antibodies (1 : 200, biotinylated immunoglobulin G, Calbiochem) for 2 hours. Sections were washed and incubated in avidin-biotin-peroxidase complex (ABC Kit, Vector) for 90 minutes, then washed with Tris-HCl (pH 7.6), and finally developed with diaminobenzidine (DAB) (1 tablet/15 mL of Tris-HCl). Next, slices were washed in PBS and mounted on histological slides. Analysis and documentation of results were performed using a Leica FW 4500 B microscope (Wetzlar, Germany). The degree of staining was provisionally graded by the following criteria: 1+, low staining; 2+, moderate staining; and 3+, intense staining detected by light microscopy ×100 augmentation (10x objective).

### 2.3. Quantitative mRNA Expression

Thawed hippocampi were homogenized in 1 mL of TRIzol reagent (Gibco BRL, Gaithersburg, MD), and total RNA was isolated according to the manufacturer's instructions. One microgram of total RNA was used for cDNA synthesis and real-time PCR gene expression analysis. Firstly, DNase I (Invitrogen) treatment at a concentration of 1 unit/*μ*g RNA in the presence of 20 mM Tris-HCl, pH 8.4, containing 2 mM MgCl_2_ for 15 min at 37°C, followed by incubation at 95°C for 5 min was performed to remove DNA contamination. Reverse transcription (RT) was carried out in a 20 *μ*L reaction in the presence of 50 mM Tris-HCl, pH 8.3, 3 mM MgCl_2_, 10 mM dithiothreitol, 0.5 mM dNTPs, and 50 ng of random primers with 200 units of Moloney murine leukemia virus reverse transcriptase (Invitrogen). This reaction was performed as follows: 20°C for 10 min, 42°C for 45 min, and 95°C for 5 min. cDNA was at that point amplified by real-time PCR on the 7500 Sequence Detection System (ABI Prism, Applied Biosystems, Foster City, CA) using the SYBR Green core reaction kit (Applied Biosystems). Polymerase enzyme was heat activated for 10 min at 95°C, 40 cycles of 15 sec at 95°C, and 1 min at 60°C amplified the transcript, and data was collected at each cycle. Experiments were performed in triplicates for each data point. Target gene mRNA expression was quantified as a relative value compared with an internal reference, GAPDH, whose expression was believed not to change between the varying experimental conditions. Rat primers used for mRNA quantification were UCP2 (GenBank accession number NM_019354.2) forward 5′-CCACAGCCACCGTGAAGTT-3′ and reverse 5′-CGGACTTTGGCGGTGTCTA-3′; Bcl2-associated death promoter (Bax) (GenBank accession number NM_017059.2) forward 5′-ACTCCCCCCGAGAGGTCTT-3′ and reverse 5′-AGTTGAAGTTGCCATCAGCAAA-3′; Bcl-2 (GenBank accession number NM_016993.1) forward 5′-GCTACGAGTGGGATACTGG-3′ and reverse 5′-GTGTGCAGATGCCGGTTCA-3′; TNF-*α* (GenBank accession number X66539) forward 5′-AAATGGGCTCCCTCTATCAGTTC-3′ and reverse 5′-TCTGCTTGGTGGTTTGCTACGAC-3′; interleukin-1b (GenBank accession number M98820), forward 5′-CACCTCTCAAGCAGAGCACAG-3′ and reverse 5′-GGGTTCCATGGTGAAGTCAAC-3′; and interleukin-6 (GenBank accession number E02522) forward 5′-TCCTACCCCAACTTCCAATGCTC-3′ and reverse 5′-TTGGATGGTCTTGGTCCTTAGCC-3′. GAPDH primers were forward 5′-TGCACCACCAACTGCTTAGC-3′ and reverse 5′-GCCCCACGGCCATCA-3′ (GenBank accession number NM_017008). A second pair of a housekeeping gene (18S rRNA, GenBank accession number Rn18s) was used to validate the results. One microliter of RT reaction was used for real-time PCR. Quantitative values for target gene and GAPDH mRNA transcription were obtained from the threshold cycle number, where the intensification in the signal associated with an exponential growth of PCR products begins to be detected. Melting curves were generated at the end of every run to confirm product uniformity. The relative target gene expression level was normalized based on GAPDH expression as an endogenous RNA control. ∆*C_t_* values of the samples were determined by subtracting the average *C_t_* value of target gene mRNA from the average *C_t_* value of the internal control (GAPDH). The 2^−∆∆*Ct*^ parameter was used to express the relative expression data.

### 2.4. UCP2 Silencing

Antisense oligonucleotide (ASO) protocol was performed using 24 Wistar male cannula-implanted rats. Briefly, rats were anesthetized with intraperitoneal injections of ketamine and xylazine (35 mg/kg and 5 mg/kg, resp.), shaved, and placed in a stereotaxic frame. Then the animal eyes were protected and hydrated with Ocry-gel. A mid-line scalp incision was performed, and the skull was exposed and cleaned of blood and periost. Subsequently, a cannula (gauge 23) was implanted unilaterally at the hippocampus according the coordinates of Paxinos et al. (AP: 3.5 mm behind the bregma, lateral: 3.1 mm, and vertical: 4.5 mm from the cerebral cortex [33]. Two screws were positioned in the skull, and each cannula was affixed into place with dental cement poured around the outer cannula and screws. A stainless steel bar extending just beyond the tip of the cannula was inserted and left in place until inoculation. All rats received ~5 mL of 0.9% saline via ip injection to rehydrate and aid in recovery from surgery. Rats were allowed 7 days postsurgical recovery before any additional experimental procedures. Pilocarpine administration was performed as previously described (*n* = 22). Five animals that received pilocarpine did not enter SE. A group that underwent *status epilepticus* (SE 5d group) was set with 7 rats, and the other 10 SE rats were subjected to ASO administration (SE ASO 5d group). Oligonucleotides were designed according to the UCP2 sequence deposited at the NIH-NCBI (NM 011671). The sequences of antisense oligodeoxynucleotides (ASO) (Invitrogen, Carlsbad, USA) were as follows: sense 5′-TGC ATT GCA GAT CTC A-3′ and antisense 5′-TGA GAT CTG CAA TGC A-3′. The effectiveness of these oligonucleotides in inhibiting UCP2 expression has been previously shown [7, 34]. Antisense oligonucleotides were dissolved in artificial cerebrospinal fluid (aCSF) immediately before administration. Microinjection (500 pmol), at a volume of 100 nL, into the bilateral hippocampal CA3 subfield was performed daily starting on the day before pilocarpine administration until 5 days post-SE. Rats that received bilateral microinjection of the same amount of aCSF (*n* = 13, being 7 pilocarpine-treated and 6 control rats) served as vehicle controls. The animals were killed 6 days after first AOS microinjection. The treatment with the sense oligonucleotides produced no variance in the expression of UCP2 as compared to control (data not shown).

### 2.5. Enzyme-Linked Immunosorbent Assay (ELISA)

UCP2, active caspase-3, phospho-AKT, IL-1*β*, and IL-6 concentrations of the rat hippocampi from control, SE, and SE ASO were quantified by ELISA. Tissues were excised and immediately frozen at −80°C. For protein extraction, the tissues were sonicated on ice in tissue extraction reagent (Invitrogen) containing protease inhibitor cocktail (Roche, Indianapolis, IN). After centrifugation at 12,000 x*g* at 4°C for 20 min, the supernatant was assayed for uncoupling protein 2 (UCP2) (Rat Mitochondrial uncoupling protein 2 ELISA kit, Cusabio®, Wuhan, China), caspase-3 activity (Caspase-3/CPP32 colorimetric assay kit, Biovision, Milpitas, CA), phospho-AKT (AKT-pS473 ELISA kit, Abcam, Cambridge, UK), IL-1*β*, and IL-6 (rat IL-1*β* or rat IL-6 Quantikine; R&D Systems, Abingdon, United Kingdom).

### 2.6. Determination of Oxidative Stress Parameters

Oxidative stress parameters were evaluated within the hippocampus homogenates of control, SE-treated, and SE ASO rats. Superoxide dismutase (SOD) activity was measured using a colorimetric commercial kit (Cayman Chemical Co., Ann Arbor, MI, USA) based on inhibition of NADH oxidation, in which superoxide radicals were generated by xanthine oxidase and hypoxanthine and detected at 450 nm using tetrazolium salt. The specific activity is represented as units per milligram of protein. Catalase activity (CAT) was measured spectrophotometrically by a commercial kit (Sigma-Aldrich, St Louis MO, USA), based on the measurement of the decomposition of H_2_O_2_. One CAT unit is defined as 1 mol of hydrogen peroxide consumed per minute, and the specific activity is reported as units per milligram of protein. MDA levels in tissue were measured spectrophotometrically as described by [35], using a commercial kit (Sigma-Aldrich, St Louis MO, USA) based on the reaction of malondialdehyde (MDA) with thiobarbituric acid (TBA), which produces a colorimetric product. MDA accumulation is indicative of the extent of cell membrane lipid peroxidation and was represented as nmol/mg protein. For the quantitative measurement of protein carbonyl content, we used a commercial kit (Sigma-Aldrich, St Louis MO, USA) based on the derivatization of protein carbonyl groups, which leads to the formation of stable dinitrophenyl (DNP) hydrazone adducts. The carbonyl content was calculated based on the molar extinction coefficient of DNPH and the results were expressed in nmol/mg protein.

### 2.7. Statistical Analysis

Data were analyzed with GraphPad Prism software 6.0 (La Jolla, CA, USA). The Shapiro–Wilk and Levene tests were used to verify normality and error variances, respectively. Two-way analysis of variance (ANOVA) complemented by Tukey's test was used to detect differences between three groups in samples with normal distribution. Unless indicated otherwise, the biochemical and molecular biology experiments were performed in triplicate. A *p* value ≤ 0.05 was considered significant. Values are expressed as means ± standard error of mean (SEM).

## 3. Results

**Figure 1:**
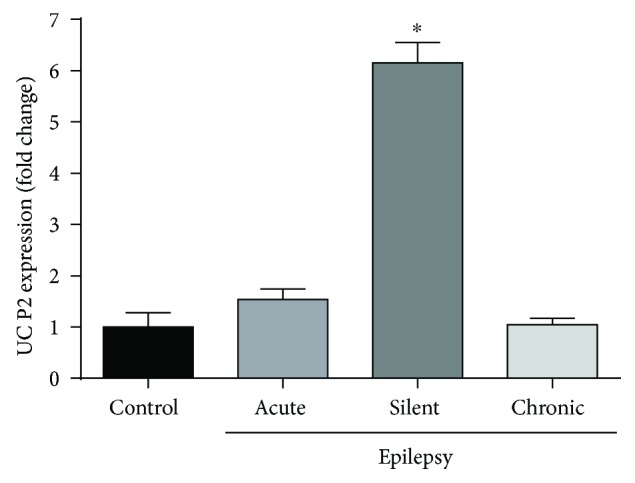
Quantitative real-time PCR for UCP2 mRNA after SE. An increased gene expression of UCP2 was found in the silent phase of the pilocarpine epilepsy model. ^∗^*p* < 0.001 according to two-way ANOVA followed by Tukey's post hoc tests. Values are means ± SEM.

**Figure 2:**
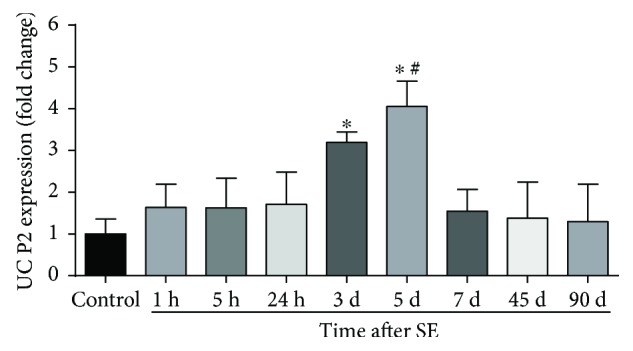
Time course expression of UCP2 mRNA after SE. UCP2 expression increases after SE in the early silent phase of pilocarpine-induced epilepsy. ^∗^*p* < 0.001 versus control, 1 h, 5 h, 24 h, 7 d, 45 d, and 90 d; ^#^*p* < 0.05 versus 3 d according to two-way ANOVA followed by Tukey's post hoc tests. Values are means ± SEM.

**Figure 3:**
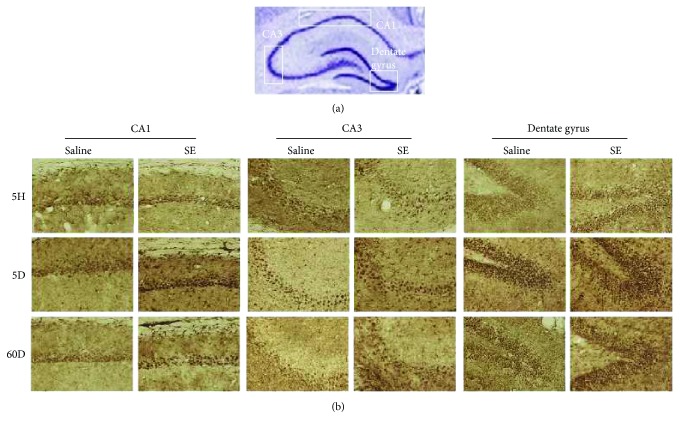
Phospho-AKT expression in the hippocampus CA1, CA3, and dentate gyrus regions of the acute (5 hours after SE (5H)), silent (5 days after SE (5D)), and chronic (60 days after SE (60D)) groups. (a) Rat hippocampal formation with Nissl staining highlighting the CA1, CA3, and dentate gyrus areas. (b) Photomicrographs of the CA1, CA3, and dentate gyrus regions of the rat hippocampus after SE processed for phospho-AKT (p-AKT) immunohistochemistry and respective controls. Pilocarpine-treated animals at the 5th day after SE (silent phase) showed higher expression of p-AKT in comparison to any other group. ×100 augmentation (10x objective).

We hypothesized that UCP2 as a neuroprotector may counterbalance the neuronal damage provoked by SE induced by pilocarpine in the experimental epilepsy model. Analyzing the hippocampus of SE rats, we noticed unaltered expression of UCP2 mRNA in the acute phase (0.80 ± 0.22) relative to vehicle-treated controls (0.52 ± 0.19). However, UCP2 mRNA expression observed in the silent phase was about 4-fold higher than that observed in the acute phase (3.26 ± 0.28). The UCP2 mRNA content in the chronic phase (0.53 ± 0.11, Figure 1) returned to basal levels and was similar to that of the control group. Both GAPDH and 18S rRNA showed similar quantification results in all real-time PCR experiments (data not shown). Therefore, we analyzed the time course expression of UCP2 mRNA during the all phases of this experimental epilepsy model. We noticed that, three days after pilocarpine injection, there was a significant upregulation of UCP2 expression (1.47 ± 0.14, Figure 2) when compared to that of control. Augmented UCP2 mRNA expression reached a peak 5 days after SE onset (3.10 ± 0.31), to further decline to baseline levels after this period. Concomitantly, we detected by immunohistochemistry robust expression of phosphorylated AKT (p-AKT), a survival cell marker, at the silent phase (5 days after pilocarpine administration) when compared to those of the control and any other experimental group (Table 1 and Figure 3).

**Figure 4:**
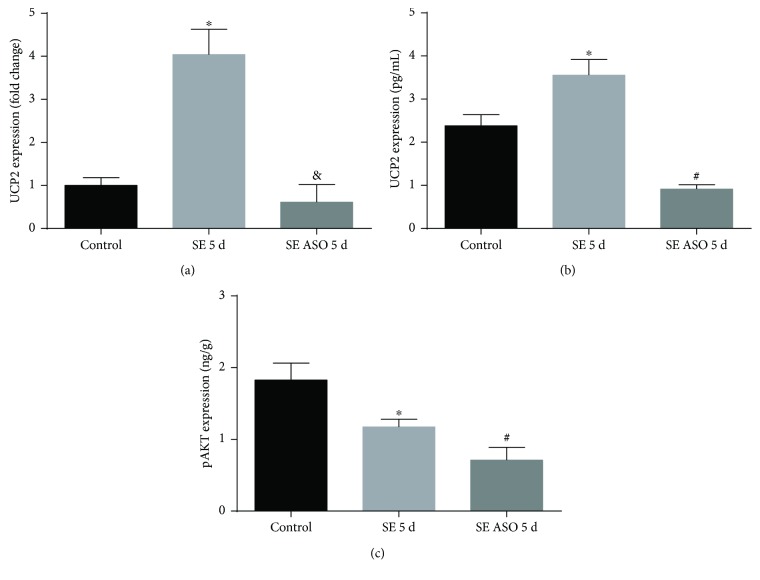
Expression of hippocampal UCP2 and p-AKT in the presence or absence of antisense oligonucleotide (ASO) treatment in SE rats. (a) Treatment with ASO diminished UCP2 mRNA expression in rats 5 days after SE onset. ^∗^*p* < 0.001 versus control and 5 d ep ASO; ^&^*p* < 0.001 versus 5 d ep; (b) UCP2 protein expression presented high levels 5 days after SE. ASO treatment successfully decreased UCP2 protein expression 5 days after SE. ^∗^*p* < 0.001 versus control; ^#^*p* < 0.001 versus control and SE 5 d; (c) p-AKT expression diminished after ASO treatment. ^∗^*p* < 0.001 versus control; ^#^*p* < 0.001 versus control and SE 5 d according to two-way ANOVA followed by Tukey's post hoc tests. Values are means ± SEM.

Treatment with UCP2 antisense oligonucleotides (ASO) has shown to effectively inhibit UCP2 generation [34]. We used the same oligonucleotides to analyze the epileptogenesis onset with UCP2 subtraction. In fact, UCP2 silencing successfully decreased UCP2 mRNA expression 5 days after SE (0.51 ± 0.21, Figure 4(a)) compared to those of the control and SE groups (0.83 ± 0.16 and 3.36 ± 0.32, resp.). ASO treatment was well tolerated and no mortality was observed in the experimental group. Rats that received UCP2 antisense oligonucleotide administration presented 100% onset of SE (i.e., all animals treated with ASO followed by pilocarpine administration developed *status epilepticus*, *n* = 10*).* In contrast, animals that received only pilocarpine were less prone to enter *status epilepticus* (7 from 12 rats). UCP2 protein quantification presented diminished values after in the SE ASO group (0.91 ± 0.21 pg/mL) compared to the SE (3.55 ± 0.21 pg/mL) and control groups (2.38 ± 0.21 pg/mL, Figure 4(a)). Furthermore, we quantified the p-AKT expression after ASO administration. SE ASO rats presented a diminished p-AKT expression (0.71 ± 0.21 ng/g, Figure 4(c)) in comparison to SE and control rats (1.17 ± 0.11 ng/g and 1.82 ± 0.34 ng/g, resp.).

**Figure 5:**
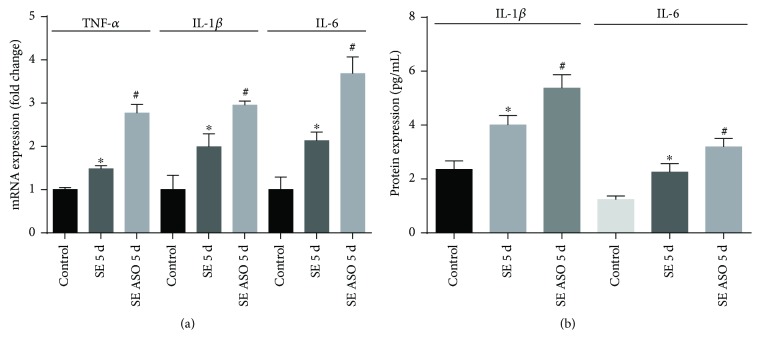
Modulation of expression of proinflammatory markers within the hippocampi with or without ASO treatment. (a) SE onset increased inflammatory marker (TNF-*α*, IL-1*β*, and IL-6) mRNA expression, while ASO treatment resulted in higher mRNA expression of these markers. (b) SE onset and ASO treatment lead to increment of IL-1*β* and IL-6 protein content. ^∗^*p* < 0.001 versus control; ^#^*p* < 0.001 versus control and SE 5 d according to two-way ANOVA followed by Tukey's post hoc tests. Values are means ± SEM.

We then analyzed the expression of inflammatory mediators within the hippocampus samples of control, SE, and SE ASO rats. We observed an augmentation of TNF-*α* (1.33 ± 0.05), IL-1*β* (2.89 ± 0.41), and IL-6 mRNA (2.48 ± 0.29) 5 days after SE onset when compared to that of control (0.90 ± 0.03, 1.40 ± 0.47, and 1.22 ± 0.39, resp.; Figure 5(a)). Nevertheless, after ASO treatment, the hippocampi of SE ASO rats presented nearly 2-fold higher expression of these proinflammatory mediators (2.49 ± 0.21, 4.13 ± 0.12, and 4.48 ± 0.47) compared to those of SE rats. Augmentation of IL-1*β* and IL-6 detected in mRNA quantification was corroborated using ELISA detection method to determine protein expression. Besides the increased expression of IL-1*β* and IL-6 in SE rats (4.01 ± 0.19 pg/mL and 2.26 ± 0.19 pg/mL, resp.), the SE ASO group showed significant and strong expression of these proinflammatory markers (5.37 ± 0.29 pg/mL and 3.19 ± 0.19 pg/mL) compared to any other experimental group (control IL-1*β*: 2.35 ± 0.13 pg/mL and IL-6: 1.23 ± 0.05 pg/mL).

**Figure 6:**
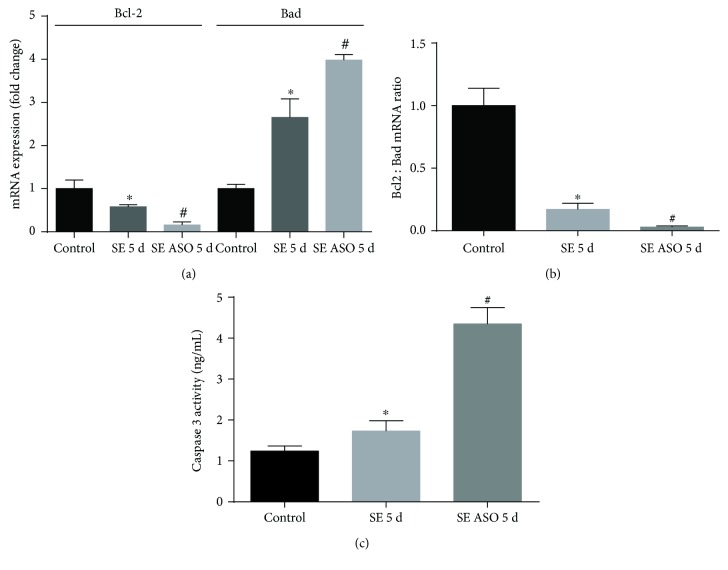
Apoptosis modulation in the presence or absence of ASO treatment in SE rats. (a) SE onset diminished bcl-2 mRNA expression, while increasing proapoptotic factor bad mRNA expression. ASO treatment increased bad expression in SE rats, with low levels of antiapoptotic factor bcl2 mRNA expression. (b) Bcl2 : bad ratio, indicating that SE followed by ASO treatment stimulated apoptosis. (c) Caspase-3 activity is increased 5 days after SE, while a marked activity augmentation was observed when SE rats were submitted to ASO treatment. ^∗^*p* < 0.001 versus control; ^#^*p* < 0.001 versus control and SE 5 d according to two-way ANOVA followed by Tukey's post hoc tests. Values are means ± SEM.

Then we analyzed the mRNA expression of apoptotic factors in the presence or absence of ASO treatment in the SE rat hippocampi in the silent phase. SE onset induced not only a reduction in antiapoptotic bcl2 mRNA expression (0.72 ± 0.21) but also a robust mRNA expression of bcl2-associated death promoter (bad) (3.58 ± 0.44), suggesting an activation of apoptosis when compared to that of control (1.95 ± 0.26 and 1.69 ± 0.32, resp.). Then we observed a strong diminished expression of bcl2 mRNA after 5 days of SE onset and ASO treatment (0.26 ± 0.05) compared to that of the SE group. The bad mRNA expression increased with SE ASO treatment (6.75 ± 0.53) compared to that of any other group (Figure 6(a)). This higher decrease in bcl2 : bad mRNA ratio suggests a more pronounced apoptotic cell death related with UCP2 silencing in the silent phase of the pilocarpine-induced epilepsy model (Figure 6(b)). To investigate that ASO treatment altered survival by increasing apoptosis, we analyzed the hippocampi for the level of active caspase-3. As shown in Figure 6(c), caspase-3 activity at day 5 was significantly higher in the hippocampus of the SE ASO group (4.35 ± 0.36 ng/mL) compared to the control (1.23 ± 0.18 ng/mL) and SE groups (1.73 ± 0.21 ng/mL), indicating augmentation of apoptosis after ASO administration.

**Figure 7:**
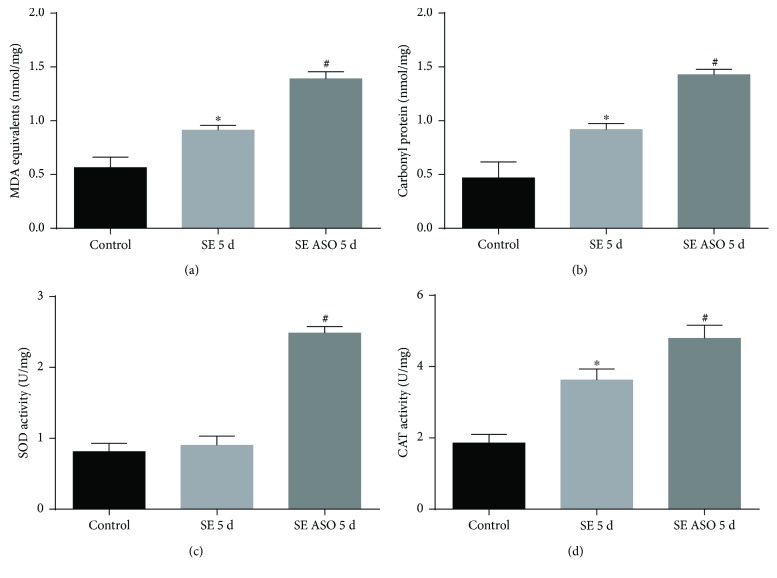
Oxidative stress within the SE rat hippocampi with and without ASO treatment. (a) MDA levels, (b) carbonyl protein, (c) SOD activity, and (d) catalase activity. ^∗^*p* < 0.001 versus control; ^#^*p* < 0.001 versus control and SE 5 d according to two-way ANOVA followed by Tukey's post hoc tests. Values are means ± SEM.

Lipid peroxidation was quantified by measuring MDA production. MDA levels were found to have increased in the hippocampus of rats that were subjected to pilocarpine administration (SE group, 0.91 ± 0.07 nmol/mg) compared to control (0.56 ± 0.15 nmol/mg). ASO treatment markedly increased the levels of MDA in the hippocampus of pilocarpine-treated rats (1.39 ± 0.11 nmol/mg, Figure 7(a)). In SE rats, the levels of carbonyl protein were found to be elevated within the hippocampus (0.92 ± 0.11 nmol/mg) compared to those of control (0.47 ± 0.23 nmol/mg). The ASO treatment increased the levels of carbonyl protein in the hippocampus of rats injected with pilocarpine (1.42 ± 0.09 nmol/mg, Figure 7(b)).

To assess the efficacy of ASO treatment on the intracellular antioxidant system, we measured ROS-related enzyme activities. The SOD enzyme activity was unaltered in the hippocampus of the SE group (0.90 ± 0.17 U/mg) compared to control (0.81 ± 0.11 U/mg). However, ASO treatment significantly increased SOD activity of SE rats (2.48 ± 0.34 U/mg; Figure 7(c)). We demonstrated that there was an increase in the levels of catalase activity (3.62 ± 0.16 U/mg) in the hippocampus after SE compared to control (1.82 ± 0.11 U/mg). It must be noted that treatment with ASO resulted in augmented CAT activity in the hippocampus (4.79 ± 0.23 U/mg, Figure 7(d)).

## 4. Discussion

In the present study, we demonstrated that rats subjected to the animal model of epilepsy induced by pilocarpine had increased expression of UCP2 in the hippocampus during the early silent phase (between 3–5 days after SE). ASO treatment successfully diminish UCP2 mRNA and protein expression in this period after SE. In addition, the brains of SE rats injected with ASO showed an increase in oxidative stress, marked by damage caused by lipid peroxidation, higher levels of protein carbonyl, and increase in the activity levels of antioxidant enzymes, SOD, and catalase. Moreover, when SE rats received ASO, we observed increase of proinflammatory marker expression and enhanced apoptosis.

Our group has reported the participation of several molecules with effects on the inflammatory process that are gradually taking an important role in epilepsy and its potential treatment [23, 25]. In order to establish mechanisms of epileptogenesis, we have observed numerous cellular and molecular changes that, with time and increasing understanding, broaden the list of new molecules that may contribute to the pathological state of the disease. While UCP2-based neuroprotection has been widely reported in many species [11, 36–44], its participation in hippocampal epilepsy needs further investigation. To this end, we observed increased expression of UCP2 after pilocarpine administration in the silent phase, reaching a peak on the fifth day after *status epilepticus* onset. This is corroborated by groups who analyzed kainic acid-induced epilepsy in rodents [43, 45, 46].

UCP2 gene expression during the silent phase suggests that this molecule might be required during epileptogenesis in neuronal cells. The silent phase of the pilocarpine-induced model is characterized by no differentiated phenotype but many metabolic alterations. We observed an increase in p-AKT in the same period in which UCP2 expression is incremented. The relation between UCP2 and AKT has already been described [11, 47], although its relevance to epilepsy remains unclear. The kinase AKT, also known as protein kinase B, is a serine/threonine-specific protein kinase that has a central role in the signaling pathways that regulate metabolism and cellular transformation. AKT can regulate cell growth positively and apoptosis negatively by activating a series of different downstream signaling molecules [48, 49]. In epilepsy, AKT phosphorylation is very welcome because they positively influence neuronal survival by reducing the cell damage observed after insult. While it does not have kinase activity, UCP2 has a close interaction with cell survival factors based on recent reports [11]. Derdák et al. [47], using a UCP2 knockout mouse model, provided the first *in vivo* evidence for a link between UCP2 and cancer, when transgenic mice showed an imbalance between epithelial cell proliferation and apoptosis.

The silencing of UCP2 gene expression is a molecular protocol targeting the mRNA of this molecule, limiting its availability by annealing between complementary nucleotide sequences. This model has previously been used with interesting results. For example, De Souza et al. [34] demonstrated that the silencing of UCP2 ameliorates the hyperglycemic syndrome in two distinct animal models of obesity and diabetes. In 2008, Degasperi et al. [7] reported that inhibiting UCP2 expression increases TNF-*α*-induced expression of markers of ROS accumulation and apoptosis. The authors postulated that induction of UCP2 expression in the rat hypothalamus could be considered an endogenous protective mechanism that might minimize the harmful effects of potent inflammatory stimuli. Our observation that rats that underwent UCP2 antisense treatment were more susceptible to *status epilepticus* might be an evidence of neuroprotection elicited by UCP2.

SE induced by pilocarpine may promote oxidative stress and could be reflected in direct activation of antioxidant enzymes. Under healthy conditions, a balance between the production of ROS and their destruction by antioxidant systems is found. Nevertheless, this balance can be altered either by increased ROS production or by a decrease in cellular antioxidant systems. Pilocarpine-induced seizures produce several changes in variables related to the generation and elimination of oxygen free radicals in adult rats [50]. SOD enzyme reaction results in H_2_O_2_ and water from dismutation of the superoxide (O_2_^−^) radical, and catalase converts H_2_O_2_ to oxygen and water [51]. Tejada et al. [52] reported that an increase in enzyme antioxidant activities was observed after SE, indicating that neurons try to counteract excessive SE-induced ROS. However, we observed an unaltered SOD activity after SE onset. SOD activity is related to mechanisms involved in the initiation and/or propagation of seizures induced by pilocarpine. This data is corroborated by another study showing unaltered SOD activity 24 h after pilocarpine treatment, suggesting that SOD activity only changes during the initiation of seizures [53].

Cao et al. [54] reported that splenocytes from UCP2 knockout mice were more susceptible to pathogen activation-induced apoptosis and that the high level of ROS in UCP2 KO mice might be the cause of the apoptotic susceptibility. In our study, when UCP2 was inhibited, the activities of antioxidant enzymes CAT and SOD were significantly increased. These results are in accordance with [7, 34] that suggested that the damaging effect of reduced UCP2 expression was linked to augmented ROS formation. Also, we showed that ASO treatment resulted in MDA and protein carbonyl augmentation in SE rats. Oxidative stress induces cell apoptosis when the endogenous antioxidant factors were decreased [7, 55]. Our results suggest that SE onset induces apoptosis in the rats' hippocampi by decreasing the antioxidant enzyme activities. The augmentation of these endogenous antioxidant systems after ASO treatment may protect the hippocampi against oxidative stress induced by pilocarpine.

Bengzon et al. [56] reported that changes in the expression and activity of cell death regulatory proteins, such as members of the Bcl2 and caspase families, occur in regions vulnerable to cell degeneration. This suggests an involvement of these factors in apoptosis following seizures. Chen et al. [57] observed that kainic acid administration led to marked neuronal apoptosis in the hippocampus, accompanied by increased levels of Bax, activated caspase-3, and decreased levels of Bcl2. In our study, bcl2 mRNA was decreased relative to bad expression after pilocarpine-induced epilepsy. Reduction of UCP2 expression results in even greater modulation of both factors, indicating more pronounced activation of apoptotic cell death when ASO are administrated, data that is corroborated by Degasperi et al. [7]. While increased caspase-3 activity was observed after SE onset, a higher augmentation was observed after SE and ASO treatment. We can then infer that neuronal cells likely had a higher susceptibility to cell death after SE. However, after ASO, this susceptibility increased. This data along with the bcl2 : bad ratio after antisense treatment highlight the neuroprotective role of UCP2. Together, these evidences demonstrate a mechanism for a protective cell defense against the insult, in accordance with several other studies [4, 44, 58].

Although the neuroprotective role of UCP2 may be dependent on the activation of several other molecules, its inhibition had been showed a decrease in the activated form of AKT, which in turn prevented AKT from exerting its catalytic activity and subsequently activating other cell survival-related molecules [59, 60]. In fact, in SE ASO rats, we observed a diminished expression of p-AKT, corroborating these findings. UCP2 is a neuroprotective factor both through its direct effects in decreasing mitochondrial ROS and through a change in the spectrum of secreted cytokines towards a more anti-inflammatory spectrum [61]. Indeed, when we reduced UCP2 expression, the proinflammatory marker expression was increased. Our data corroborate a study using neurotoxin MPTP-treated UCP2-deficient mice that also showed an enhanced expression of proinflammatory cytokines such as tumor necrosis factor-*α* and interleukin 1*β* [62].

## 5. Conclusion

Our data suggest that UCP2 expression can modulate apoptotic responses and ROS formation to alleviate cellular damage in the hippocampus generated by an excessive neuronal insult as long-lasting SE induced by pilocarpine. Although further studies are needed to identify the molecular pathways of epileptogenesis, our results contribute to the increasing knowledge about the activity of the UCP2-survival-apoptosis axis in epilepsy.

## Figures and Tables

**Table 1:** Immunoreactivity for phospho-AKT in the CA1, CA3, and dentate gyrus in an epilepsy model induced by pilocarpine administration.

Hippocampal formation	Control (saline)	Acute (5H)	Silent (5D)	Chronic (60D)
CA1	+	+	+++	++
CA3	++	+	+++	+
Dentate gyrus	+	+	+++	++

Staining of phospho-AKT was scored by two independent observers as follows: +: low; ++: moderate; +++: high intensity based on photomicrographs presented in Figure 3. Control: saline-treated rats; acute: 5 hours after *status epilepticus* (SE); silent: 5 days after SE; chronic: 60 days after SE.

## Data Availability

The data used to support the findings of this study are available from the corresponding author upon request.
